# The evaluation of non-enzymatic antioxidants effects in limiting tumor- associated oxidative stress, in a tumor rat model

**Published:** 2015

**Authors:** R Grigorescu, MI Gruia, V Nacea, C Nitu, V Negoita, D Glavan

**Affiliations:** * ”Carol Davila” University of Medicine and Pharmacy, Bucharest, Romania; ** ”Prof. Dr. Alex. Trestioreanu” Oncological Institute, Bucharest, Romania

**Keywords:** non-enzymatic antioxidants, oxidative stress, malignancy, Walker carcinoma 256

## Abstract

Active oxygen species are produced as a consequence of normal aerobic metabolism. Of these, free radicals are usually metabolized or inactivated in vivo by a team of antioxidants. Individual members are a trained team fighting antioxidants to prevent the generation of ROS, destroy or oxidizing potential of capture. In terms of physiological oxidative stress, induced tissue attack is minimal. A relative or absolute deficiency in the antioxidant defense may lead to increased oxidative stress and this event is associated with both the causes and consequences of diseases and cancer, included here. The aim of the study is to identify the role of antioxidant defense systems and the reduction of oxidative stress in dynamic growth and development of malignant tumors.

Our in vivo study was developed and referred to carcinosarcoma carriers Wistar rats treated with non-enzymatic antioxidants: vitamin C, vitamin A, zinc salt (II), and arginine in various combinations. Treatment was initiated three weeks before tumor induction.

## Introduction

The role of reactive oxygen species and free radicals in human pathology is now widely accepted. Special interest to the scientific community for reactions occurring in reactive oxygen species (ROS) is supported not only by the large number of processes that occur but also for their diversity which is absolutely amazing. ROS are formed continuously in cells in various metabolic processes, sources of training being either endogenous or exogenous. Drugs, ultraviolet radiation, air pollution, chemical agents may induce overproduction of ROS in cells [**1**].

Production of active oxygen metabolites is a physiological process that grows ever stronger with age, inflammatory processes, infectious diseases, exposure to radiation, chemical pollutants, a/ lifestyle, acute or chronic, a longer period of time.

Once formed, ROS are rapidly decomposed by antioxidant enzyme systems and non-enzymatic systems existing in cells.

When they are overwhelmed by excess production of active species, it is the so-called “oxidative stress” fundamentals of researchers from USA, UK, Germany (Ames B [**2**], Halliwell B [**3**], Himself H [**4**], which stress to be regarded as a dynamic imbalance, that can be defined as a pathological change caused by the action of a strong flow of active oxygen metabolites, in turn caused by an imbalance between their production systems and the endogenous antioxidant protection.

In other words, to allow a complex phenomenon involving antioxidants, Gutterige Halliwell [**5**] proposed the following definition: antioxidant is any substance whose presence in small quantities compared with oxidizable substrate, significantly delays or prevents oxidation of that substrate”. The term “substrate oxidizable” includes any molecule that exists in living organisms. The definition highlights the importance of “target lesion” study and the source of reactive species used when investigating the in vitro antioxidant action.

Healthy body reacts with a high efficiency to produce ROS, because biological antioxidant system appeared on the evolutionary scale of beings, even bacteria, which proved important fundamental scientific and active oxygen metabolites living organisms [**6**].

Biological antioxidant system includes enzymatic systems and endogenous non-enzymatic systems able to capture or decompose reactive metabolites of oxygen. Epidemiological studies showed that people who consume a diet rich in vitamin E, β-carotene and vitamin C, present a low risk of cancer or other diseases associated with ROS, compared with those who have a diet deficient in these vitamins. Beneficial effect is installed but when the diet contains a greater variety of antioxidants and not only large amounts of the above mentioned [**7**,**8**].

The book follows the modulation of endogenous antioxidant activity by using antioxidant compounds on reactive oxygen species associated with malignant growth and development.

## Materials and methods

Biological material included Wistar albino rats obtained from Biobase Institute, cared and fed the standard diet that provided all the nutrients the rodents needed. Followed daily, in order to be placed in the experimental groups, only healthy animals that had similar characteristics (gender, weight, age) for batch homogeneity, were included in the study. Principles and rules of ethics in scientific research such as experimental plots were chosen to reduce the maximum number of individuals; there was a justification for their reduction (reduction principle) and maneuvers that were chosen to minimize the pain caused (pain limiting principle), were performed.

Studies were conducted on experimental tumor bearing animals, such as Walker 256 carcinosarcoma [**9**]. Carcinomatous variant of the tumor, selected during passages, was made of cellular structures supported by stromal tissue composed of reticulin and collagen fibers. The tumor was well vascularized, with dilated vessels near the numerous outbreaks of necrosis.

Sarcomatous cell variant showed fusiform cells surrounded by reticulin and collagen fibers which were less numerous. Sarcomatous component of the tumor had a predilection for the vicinity of surface necrosis.

The percentage trapping of tumor graft is of 80-90% with seasonal changes. Tumor outlet varies with age and sex of animals. Walker 256 tumor maintained Wistar rats by subcutaneous grafts rarely metastasize, and loco-regional lymph invasion occurs in terminal tumor growth when tumor necrosis phenomena are emphasized. Ascitic form and converting solid shape can be maintained in ascitic form by serial passages in rats.

Tumor is a standard used in preclinical screening of antitumor substances, as well as in various experimental models of chemo-radio-immunotherapy, but also in lymph node metastasis patterns obtained by open graft technique. Tumor is also widely used as experimental model in rats to induce cachexia; cachexia syndrome is a major cause of cancer mortality.

**Fig. 1:**
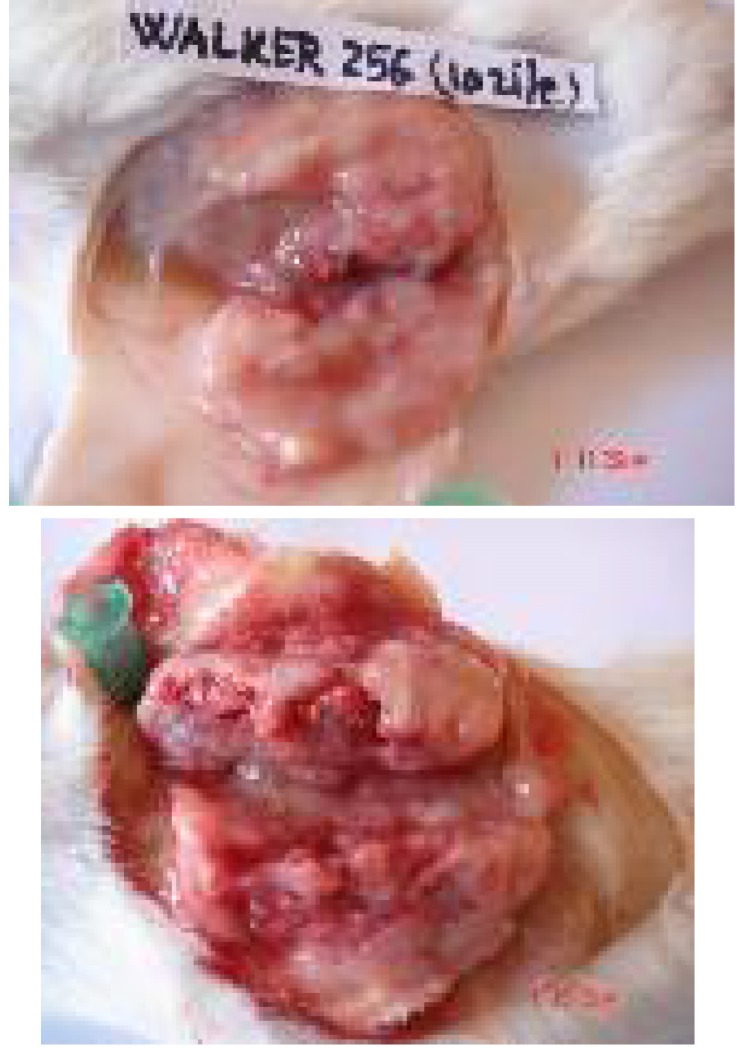
Walker carcinoma 256 The tumor is well vascularized, with dilated vessels near many centers of necrosis.

Experimental Model: The administration of antioxidants was performed on three groups of animals compared with untreated control group, daily food bowl placed in the three weeks before tumor graft.

Group 1 was treated with vitamin A 3000 IU/ kg, 1 g/ 70 kg body + vitamin C, zinc ions 30 mg/ kg, group 2 the same reference dose zinc ions, group 3 treated with vitamin A, vitamin C + + Zn salt, arginine.

Doses are equivalent to the human body, calculated per kilogram body weight were collected biological samples consisting of whole blood and tumor tissue at 5, 10, 15 days after dosing and at 7.14, and 21 days after inoculation of tumor cells for all groups investigated.

Biochemical parameters investigated: was the index of lipid peroxidation, assessed by measuring serum malondialdehyde concentration, the end product of degradation of lipid hydroperoxides, measured by reaction with 2-tiobarbituric acid [**10**], albumin thiols Schosinski t measured by the method [**11**] by using Ellman reagent in a measurable reaction spectrophotometrically at 412 nm. Total antioxidant serum is measured by the ability to reduce iron (III) to iron (II) [**12**]. At low pH Felll complex - tripyridyl s triazine (Felll-TPTZ) is reduced to properly complex Fe (II) complex intense blue color, measurable, with a maximum absorption at 593 nm. Any response with positive redox potential mentioned conditions may reduce the TPTZ-complex. In Felll complex, Fe (II)-TPTZ uses excess salt Fe (III) complex of Felll-limiting factor and the formation TPTZ color reduces the ability of the sample. Measured response to the reduction of ferric iron complex, colored reaction is monitored for four minutes, measuring the optical density at 593-nm.

## Results and discussion

**Fig. 2:**
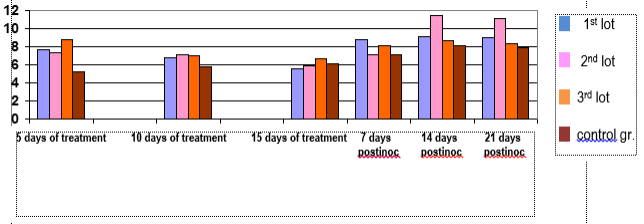
Values determined in the dynamics of lipid peroxides and antioxidant treatment in experimental tumor induction

It shows a decrease of lipid peroxidation reaction during treatment with antioxidants investigated in all groups compared to the control group. After cessation of exogenous administration of compounds to induce experimental tumor growth rate, the result was the formation of reactive oxygen species that initiated a reaction of lipid peroxidation. This increase shelf remaining when tumor size reached the 21-day was related to the tumor registry. The remaining in the plateau of high values associated with a lower substrate concentration and a reaction specific to polyunsaturated fatty acids. Exogenous compounds in the feed, given daily until graft, did not provide an antioxidant protection and in tumor growth and development. Most protection was provided by a complex containing antioxidants and arginine.

**Fig. 3:**
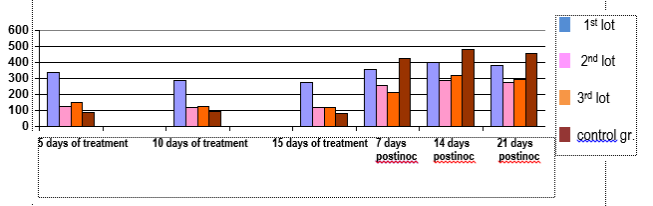
The values of total thiols measured in serum of animals bearing tumors

Regarding these developments, thiols reduce both dynamic and growing treatment during tumor development, which is also expected to change (tumor-generating oxidative stress). Towards the end of the experiment, as recovery brought “deposits” from the liver, it took a sharp increase in serum thiols, because it greatly increased reduced glutathione in tumors.

**Fig. 4:**
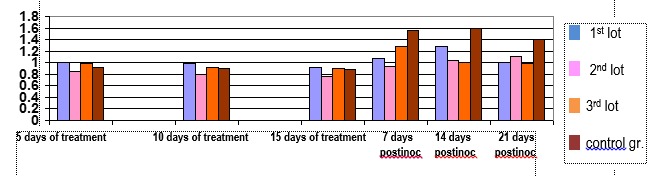
Total antioxidant concentration measured by the reduction reaction of iron (III)

It showed a normal storage in total antioxidant levels during the administration of exogenous antioxidants and antioxidant escape supervision of oxidative stress during tumor growth and development.

The results of these determinations especially emphasized that group 2, receiving only needs to zinc ions (II) had significantly higher antioxidant reactions, suggesting that the association of zinc ions with vitamin C and A augmented by arginine could activate antioxidant defenses and reduce oxidative stress more effectively.

**Determining the parameters of oxidative stress in tumor tissue**

**Fig. 5:**
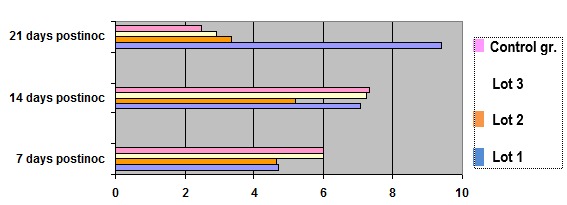
Lipid peroxide values in tumor growth dynamics, determined in crude tissue homogenate

Tumor tissue showed a steady decrease of peroxidation in tumor development dynamics for the control group and a slight decrease for the treated groups, suggesting that treatment is poor as far as management is concerned. Peroxidation reaction is a chain reaction, indicating that substrate consumption decreased, but the lack of antioxidant protection did not decrease. The danger is the fact that reactive oxygen species, especially peroxides can move from place, can initiate the reaction and other reactions of healthy tissue surrounding normal cells. Especially affecting the structure of links double membranes, they can then modify the initial structure and functionality; the loss of stiffness, modified membranes are more fluid, expressed on their surface receptors and have another antigenicity modifying the surface.

**Fig. 6:**
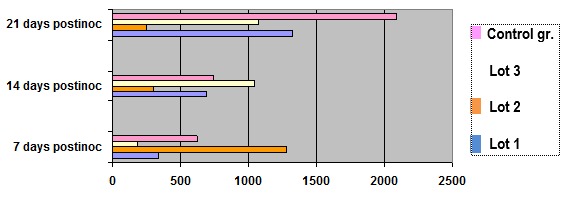
Thiols level recorded in tumor tissue

**Fig. 7:**
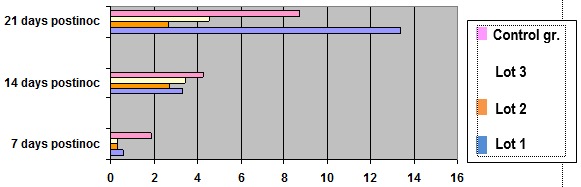
Total antioxidant values in tumor tissue in experimental tumor growth dynamics

Tumor Registry definitely creates a significant oxidative stress, so that it was expected to follow an ascending line. Three weeks before the graft, antioxidant administration probably confers only limited protection, which is insufficient.

## Discussion

It is known that in vivo test results are not always predictable because of many factors (sometimes hardly controllable) that may influence the experiment: individual variability, state enzyme systems, pathological condition, some inflammatory processes which can occur during testing, biorhythms (to obtain differences between the spring-summer tests than those made in autumn or winter).

One can appreciate that constant administration of antioxidants could be beneficial for the body [**13**].

The results in this paper demonstrate the role of exogenous antioxidants in reducing oxidative stress especially when they are maintained in a complex mixture that contains vitamins A and C, zinc ions and arginine. It was also demonstrated that the tumor induces oxidative stress; therefore, it can only decrease with an increased intake of antioxidants. For this purpose, administration may be beneficial for a longer period of time or a higher concentration, but a calculated caution measure should be taken so that they do not exceed the limit of beneficial antioxidants and become pro-oxidants.

Since many oxidizing agents are first generated in aqueous phase, water-soluble antioxidants can be considered the first line. On the other hand, lipid antioxidants are more effective. Antioxidants in aqueous phase probably act as primary scavengers, toxic metabolites reacting directly with oxygen to form more stable compounds [**14**]. While they may be protective against lipid peroxidation and the degree of protection against a specific molecule depending on the depth of that part of the lipophilic molecule, which is anchored to the lipid membrane [**15**]. There is a synergic cooperation of bio-antioxidants at the cellular level.

Epidemiological studies showed that people who consume a diet rich in vitamin E, β-carotene and vitamin C, present a low risk of cancer or other diseases associated with ROS, compared with those who have a diet deficient in these vitamins. A beneficial effect can install, but when the diet contains a variety of antioxidants and not only large amounts of the above mentioned.
